# Diagnostic value of galactomannan in tracheobronchial aspirate for *Aspergillus* infection in lung transplant recipients (the GALACTBAS study)

**DOI:** 10.1128/jcm.01556-25

**Published:** 2026-04-30

**Authors:** Arnau Monforte, Maria Teresa Martín-Gómez, Cristina Berastegui, Ester Márquez-Algaba, Judith Sacanell, Joel Rosado, Anna Falcó-Roget, Gonzalo Escudero, Jose Casanovas, Cristina Kirkegaard-Biosca, Berta Sáez-Giménez, Víctor Monforte, Joan Gavaldà, Oscar Len, Ibai Los-Arcos

**Affiliations:** 1 Infectious Diseases Department, Hospital Universitari Vall d’Hebron, Vall d'Hebron Research Institute (VHIR), Vall d’Hebron Barcelona Hospital Campus 203275, Barcelona, Spain; 2 Department of Medicine, Universitat Autònoma de Barcelona 16719, Barcelona, Spain; 3 Microbiology Department, Hospital Universitari Vall d’Hebron, Vall d'Hebron Research Institute (VHIR), Vall d’Hebron Barcelona Hospital Campus 203275, Barcelona, Spain; 4 Pneumology Department, Hospital Universitari Vall d’Hebron, Vall d'Hebron Research Institute (VHIR), Vall d’Hebron Barcelona Hospital Campus 203275, Barcelona, Spain; 5 CIBER de Enfermedades Respiratorias (CIBERES), Instituto de Salud Carlos III 38176, Madrid, Spain; 6 Intensive Care Department, Hospital Universitari Vall d’Hebron, SODIR Research Group, Vall d'Hebron Research Institute (VHIR), Vall d’Hebron Barcelona Hospital Campus 203275, Barcelona, Spain; 7 Thoracic Surgery Department, Hospital Universitari Vall d’Hebron, Vall d'Hebron Research Institute (VHIR), Vall d’Hebron Barcelona Hospital Campus 203275, Barcelona, Spain; 8 CIBER de Enfermedades Infecciosas (CIBERINFEC), Instituto de Salud Carlos III 38176, Madrid, Spain; University of Calgary, Calgary, Alberta, Canada

**Keywords:** fungal infection, lung transplantation, invasive aspergillosis, pulmonary aspergillosis, fungal biomarkers

## Abstract

Lung transplant recipients (LTR) are highly susceptible to *Aspergillus* infection (AI), but diagnosis may be limited by the invasiveness of bronchoalveolar lavage fluid (BALF) collection. Proximal airway samples may provide a less invasive alternative. We conducted a retrospective single-center study including all adult LTR (March 2018–March 2022) who underwent bronchoscopy with tracheobronchial aspirate (TBA) and BALF galactomannan (GM) testing in the same procedure. They were processed for mycological culture and EIA-based GM (Bio-Rad) after cysteine homogenization. AI was defined according to ISHLT consensus. A ROC curve for TBA GM and its diagnostic performance were assessed. A total of 545 paired TBA–BALF samples from 282 LTR were analyzed. Proven or probable AI was diagnosed in 22 samples (4%). The optimal TBA GM cut-off was ≥0.54 optical density index (ODI), yielding 95.2% sensitivity, 92% specificity, and an AUC of 0.97 (95% CI, 0.95–0.99). In comparison, BALF GM at ≥1.0 ODI showed 33.3% sensitivity and 99.6% specificity (AUC 0.88, 95% CI, 0.80–0.95) although BALF GM values were significantly higher in invasive infections compared with non-invasive infections (1.49 vs 0.14; *P*-value = 0.007). TBA cultures were more frequently positive than BALF (24% vs 9.9%). Finally, TBA and BALF GM values demonstrated moderate correlation (*r* = 0.54), and combined testing with concordant results improved accuracy to 99.6%. TBA GM demonstrated higher sensitivity than BALF GM for detecting AI in LTR, while maintaining high specificity. Proximal airway samples could complement BALF in future diagnostic strategies.

**IMPORTANCE:**

This study provides evidence that galactomannan antigen testing (GM) in tracheobronchial aspirate (TBA) is a highly sensitive, reliable, and less invasive diagnostic tool for detecting Aspergillus infection in lung transplant recipients under universal antifungal prophylaxis, compared to the current standard bronchoalveolar lavage fluid (BALF) GM. These findings highlight the diagnostic value of proximal airway specimens, consistent with the pathophysiology of post-transplant aspergillosis, which often originates in the bronchial tree and may not be adequately captured by BALF early in the disease course. Importantly, the study shows that combining TBA and BALF GM results yields near-perfect diagnostic accuracy, supporting a complementary diagnostic strategy rather than a replacement approach. Although this is a proof-of-concept study, it fills a critical evidence gap in transplant infectious diseases and lays the groundwork for safer, more accurate, and earlier diagnosis of Aspergillus infection, with potential implications for clinical practice, antifungal stewardship, and future guideline development.

## INTRODUCTION

Fungal infections, particularly aspergillosis, pose a significant threat to lung transplant recipients (LTR) due to their severely compromised immune systems. Accurate and timely diagnosis of these infections is crucial for effective management and improved patient outcomes, but it remains challenging (1). Depending on the guideline, current definitions of proven invasive aspergillosis include fungal culture of appropriate biological material and/or histopathological confirmation although these methods have limited sensitivity (30%–50%) and require considerable time to yield results (2–4).

Galactomannan (GM) antigen detection in bronchoalveolar lavage fluid (BALF) plays a central role in supporting *Aspergillus* infection (AI) diagnosis (3, 5, 6), and high optical density index (ODI) values have been associated with poorer outcomes (7). It provides faster results and higher sensitivity than fungal culture. Given that the respiratory tract is the primary portal of entry for *Aspergillus* spp., GM testing in respiratory samples is considered more sensitive than serum testing, particularly in non-neutropenic individuals and outside the context of influenza-associated disease (8–12). Several studies have analyzed its diagnostic value specifically in LTR (10, 13–18); however, the optimal cut-off value is not entirely defined. Current EORTC (3) and ISHLT (5) guidelines suggest the cut-off of ≥1, while ESCMID-ECMM (6) recommends 0.5 to 1.0.

Nonetheless, obtaining BALF can be invasive and challenging, particularly in critically ill patients with severe respiratory insufficiency, and requires appropriate training and expertise. Some studies explored samples from proximal lower airway through non-directed bronchial lavage, showing similar performance than BALF (19–21). The SARS-CoV-2 pandemic also provided the opportunity to study less invasive respiratory samples for the detection of COVID-19-associated pulmonary aspergillosis, yielding promising results (22–24). Galactomannan testing in tracheobronchial aspirate (TBA) could provide similar accuracy than BALF for diagnosing AI, especially in the proximal lower airways. However, no data are currently available in LTR, a population particularly prone to bronchial infections.

We hypothesized that GM in TBA could complement BALF GM. The main outcome of the study was to evaluate sensitivity, specificity, positive predictive value (PPV), negative predictive value (NPV), and likelihood ratios (LR) of GM testing in TBA for diagnosing AI in LTR. Secondary objectives were to assess correlation between TBA and BALF GM, the diagnostic performance of combined tests, variation of ODI values by infection type, and performance in the presence of non-*Aspergillus* fungi.

## MATERIALS AND METHODS

An observational retrospective single-center study was performed at Hospital Universitari Vall d’Hebron (Barcelona, Spain). All consecutive adults (≥18 years) who received a lung transplant from March 2018 to March 2022 and who underwent at least one bronchoscopy with GM testing in BALF and TBA, were included. In cases where AI was diagnosed, only samples from the first bronchoscopy were analyzed to avoid the potential effect of the added antifungal treatment on the GM value. Data were collected from the electronic medical records. Variables included demographic characteristics, transplant-related variables, and post-transplant complications including AI. Microbiological tests from each bronchoscopy performed were also collected from microbiology databases, including fungal culture and GM testing in both TBA and BALF. Serum GM was ordered along the diagnostic bronchoscopy according to the treating physician. GM values from serum samples obtained within 72 h before or after the bronchoscopy were also recorded. Analyses were based on available cases, and missing values were not imputed. This study was approved by the hospital ethics committee (PR(AG)259/2018) and followed the ethical principles based on the latest version of the Declaration of Helsinki. The requirement for informed consent was waived owing to the retrospective nature of the study. All data were handled in accordance with institutional and national data protection regulations.

All LTRs received lifelong prophylaxis with nebulized liposomal amphotericin B (n-LAB) according to protocol: 25 mg (6 mL) three times per week during the first 90 days post-transplant, once weekly from days 90 to 180, and every 2 weeks thereafter. Surveillance bronchoscopy was performed on all LTRs before hospital discharge after the lung transplant procedure, and whenever a decline in forced expired volume (FEV1) or symptoms or signs of infection were present. In LTR with bronchial stenosis or fistula, a therapeutic bronchoscopy could be required for balloon dilations or the placement of a bronchial stent. According to our center protocol, TBA was performed during the bronchoscopy procedure by suctioning secretions from the trachea up to the bronchial tree, including the bronchial suture area. Afterward, BALF was obtained by instilling 90 mL of normal saline into the distal airway, which was then suctioned.

Fungal culture and GM testing were performed per protocol in every BALF and TBA since March 2018. Direct microscopic examination was performed on all samples and interpreted in conjunction with the remaining diagnostic tests. Positive microscopic findings or high GM levels with negative culture usually prompted additional molecular testing. Direct microscopic examination was not systematically recorded in a structured manner in the data sets used for this retrospective analysis and, therefore, was not included in the study endpoints. TBA GM testing was performed in an exploratory context within an institutional quality-improvement initiative aimed at assessing the diagnostic performance of proximal airway testing and was continued following encouraging results observed during the SARS-CoV-2 pandemic. Therefore, results were not initially available to treating physicians due to the absence of validation. This approach allowed a retrospective assessment of the diagnostic performance of TBA GM while minimizing incorporation bias. GM testing was performed using the Platelia *Aspergillus* Ag enzyme immunoassay (Bio-Rad, Marnes-la-Coquette France). An optical density index (ODI) cut-off of ≥0.5 for serum samples was used following manufacturer’s instructions and ≥1.0 for BALF according to current recommendations in LTR (5).

Cases were classified as *Aspergillus* infection (AI), *Aspergillus* colonization, and no AI or colonization according to ISHLT criteria (4). In infections or colonizations, *Aspergillus* spp. were detected in respiratory secretions by fungal culture, or a positive GM in BALF. TBA GM results were not considered in the definition of cases. Proven AI diagnosis relied on histologic changes consistent with fungal invasion of the tissue. Probable AI required the presence of symptoms, endobronchial lesions in tracheobronchitis or radiologic changes in pneumonia. Non-invasive AI was non-ulcerative tracheobronchitis and bronchial stent infection, where tissue damage was not demonstrated. Invasive AI included anastomotic infection, ulcerative tracheobronchitis, and *Aspergillus* pneumonia, with evidence of tissue damage observed through bronchoscopy, radiology, or biopsy. In the absence of symptoms, radiologic, and endobronchial changes despite the *Aspergillus* spp. isolation or positive GM in BALF, the episode was defined as a colonization. All cases of colonization or AI were independently reviewed by two infectious diseases physicians, and any discrepancies were resolved through consensus with three additional physicians.

### Statistics

Quantitative variables were reported using median and interquartile range. Qualitative variables were shown as absolute value and percentage. Kruskal-Wallis test was applied for non-parametric quantitative variables. The area under the receiver operating characteristics (ROC) curves was generated to assess the diagnostic performance of TBA GM, and a cut-off value was established according to the Youden Index. Sensitivities, specificities, positive and negative likelihood ratios (LR) were reported. Also, PPV and NPV were calculated for AI prevalences of 5% and 10%. Pearson correlation was used to assess concordance between BALF and TBA GM values. A *P* value < 0.05 was considered significant. Data analyses were performed with Stata 18.0 (StataCorp. 2023. Stata: Release 18. Statistical Software).

## RESULTS

A total of 1,183 bronchoscopy samples were screened. Excluded samples were 622 lacking both TBA and BALF specimens and 16 that were obtained under systemic antifungal treatment following an AI diagnosis. In the end, 545 paired TBA and BALF samples, obtained from the same bronchoscopic procedures in 282 LTR, were included in the study. The demographic and clinical characteristics of the LTR are summarized in Table 1. A median of 1 bronchoscopy (IQR 1–2) was performed per each LTR. All TBA and BALF samples underwent both fungal culture and GM testing, and the median time from the lung transplant procedure to the bronchoscopy was 44.5 days (IQR 15–258.3).

**TABLE 1:** Baseline characteristics of LTR^*a*^

Baseline characteristics		Number of patients
LTR		282
Age at transplant, years (IQR)		58.8 (49.5–63.8)
Women		123 (43.6)
Transplant indication		
Interstitial lung disease		146 (51.8)
COPD		74 (26.2)
Pulmonary hypertension		17 (6.0)
Cystic fibrosis		15 (5.3)
Bronchiectasis		11 (3.9)
CLAD		6 (2.1)
Other		13 (4.6)
Single lung transplant		30 (10.6)
Pretransplant *Aspergillus* colonization		16 (5.7)
Lifelong prophylaxis with n-LAB		282 (100)
Acute rejection		141 (50.0)
Bronchial stenosis		22 (7.8)
Bronchial stent or prosthesis		13 (4.6)
Number of bronchoscopies per LTR		1 (1-2)
*Aspergillus* infection		22 (7.8)
Non-invasive	Tracheobronchitis	9 (3.2)
	Bronchial prosthesis infection	1 (0.4)
Invasive	Anastomotic infection	2 (0.8)
	Ulcerative tracheobronchitis	2 (0.8)
	Pneumonia	8 (2.8)

^*a*^ Data are expressed as numbers (%) unless otherwise indicated. CLAD, chronic lung allograft dysfunction; COPD, chronic obstructive pulmonary disease; LTR, lung transplant recipients; n-LAB, nebulized liposomal amphotericin B.

Of the 545 samples, 22 (4%) originated from distinct LTR with proven (*n* = 3; 13.6%) or probable AI (*n* = 19; 86.4%). The remaining 523 samples (96%) were classified as negative for AI and included cases of *Aspergillus* colonization, isolation of other fungal species, or no fungal growth.

**Fig 1:**
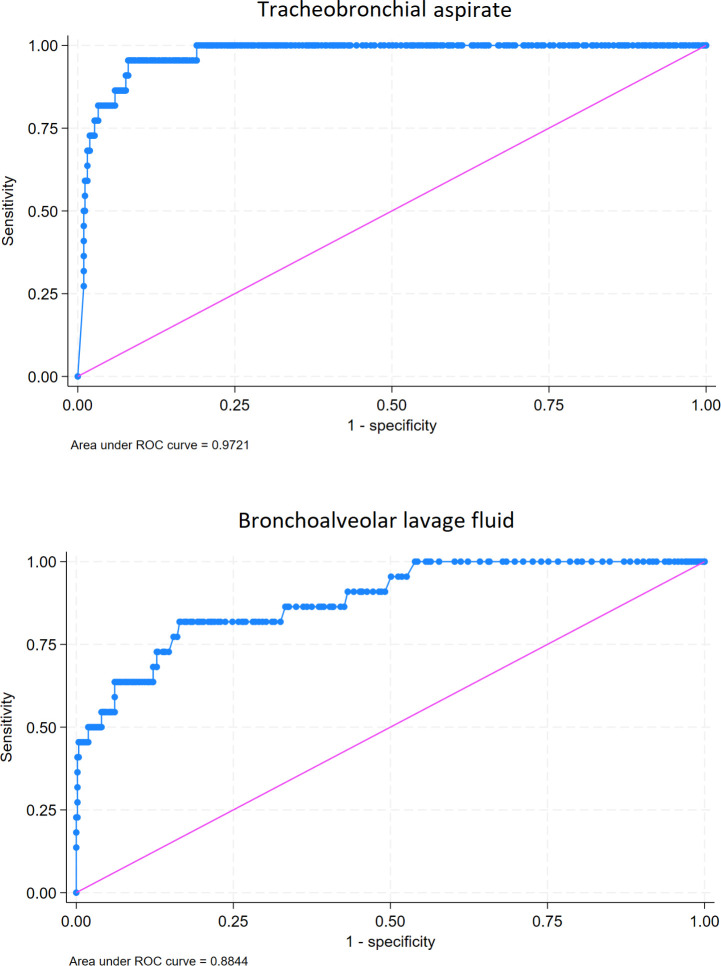
ROC curve for TBA and BALF GM. BALF, bronchoalveolar lavage fluid; GM, galactomannan; ROC, receiver operating characteristics; TBA, tracheobronchial aspirate.

An optimal cut-off ODI value of ≥0.54 for TBA GM to predict AI was identified, yielding a sensitivity of 95.2%, specificity of 92%, overall accuracy of 92.1%, and an area under the ROC curve (AUC) of 0.97 (95% CI, 0.95–0.99). Positive and negative LR were 8.91 and 0.05, respectively. At TBA GM ≥1.0, diagnostic performance remained strong (sensitivity 81.8%, specificity 95.0%); however, ≥0.5 yielded better results according to the ROC curve. For BALF GM, sensitivity was 33.3% when using the ≥1.0 ODI cut-off with 99.6% specificity. A ≥0.5 ODI cut-off improved sensitivity to 42.9% with similar specificity (98.7%). The AUC was 0.88 (95% CI, 0.80–0.95) (Fig. 1). Positive and negative predictive values varied according to the cut-off threshold and the infection prevalence (Table 2).

**TABLE 2:** Accuracy of GM ODI breakpoints in TBA and BALF for the diagnosis of *Aspergillus* infection^a^

GM ODI		Se	Sp	LR+	LR-	5% prevalence of AI		10% prevalence of AI	
						PPV	NPV	PPV	NPV
TBA (*n* = 545)	≥0.2	100	72.08	3.58	0	15.89	100	28.51	100
	≥0.5	95.24	90.44	8.91	0.05	34.90	99.72	53.09	99.42
	≥0.8	81.82	94.46	14.75	0.19	42.65	98.95	61.09	97.80
	≥1.0	81.82	95.03	16.46	0.19	47.18	98.96	65.35	97.83
BALF (*n* = 545)	≥0.2	61.90	93.13	9.01	0.41	32.17	97.89	50.03	95.65
	≥0.5	42.86	98.66	32.08	0.58	62.73	97.04	78.04	93.95
	≥0.8	42.86	99.43	74.85	0.57	79.83	97.06	89.31	94
	≥1.0	33.33	99.62	87.33	0.66	82.19	96.60	90.69	93.08
Concordant TBA + BALF (*n* = 484)	≥0.5 BA and ≥1.0 BALF	87.5	99.79	416.5	0.13	95.64	99.35	97.88	98.63

^a^ Approximation to the optimal TBA cut-off (0.54, Youden Index 0.87). AI, *Aspergillus* infection; BALF, bronchoalveolar lavage fluid; GM, galactomannan; LR+, positive likelihood ratio; LR−, negative likelihood ratio; NPV, negative predictive value; ODI, Optical Density Index; PPV, positive predictive value; Se, sensitivity; Sp, specificity; TBA, tracheobronchial aspirate.

**Fig 2:**
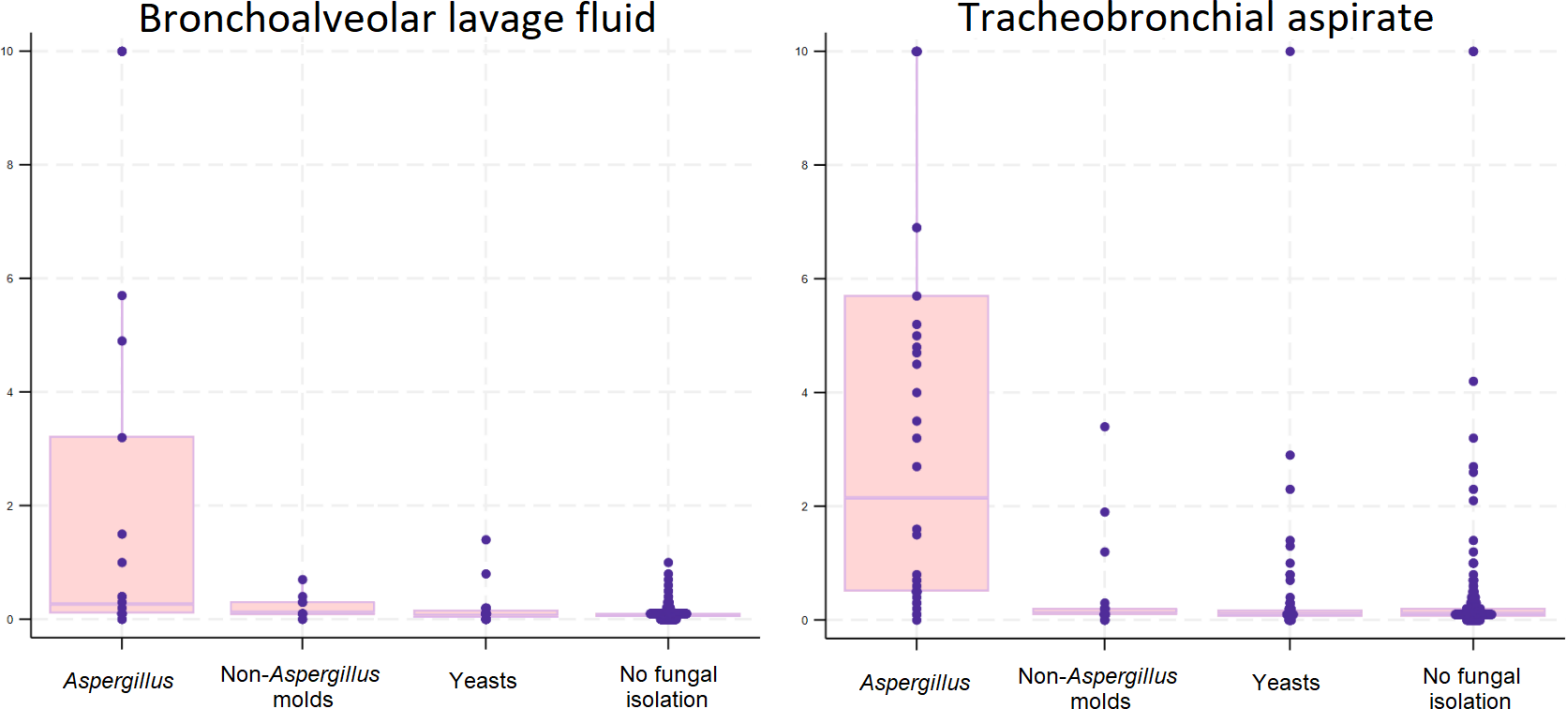
GM ODI value according to the corresponding fungal culture in BALF and TBA. GM, galactomannan; ODI, optical density index.

Positive fungal cultures were obtained in 24% of TBA samples and 9.9% of BALF samples. There was 81.5% concordance between the two samples: 38 (7%) recovered the same fungal species, and 406 (74.5%) were both negative (Table S1). Significantly higher GM values were obtained in *Aspergillus* spp. culture positive TBA and BALF samples as compared to other molds, yeasts, or samples with no fungal growth (Fig. 2). Only one participant with a *Purpureocilium lilacinus* isolation without *Aspergillus* spp. had a positive TBA GM (1.88 ODI).

In both TBA and BALF, AI was also associated with significantly higher GM values than samples without infection. TBA GM ODI were similar between invasive and non-invasive infections (6.32, IQR 3.97–10 vs 3.83, IQR 0.63–4.48; *P*-value = 0.065). In contrast, BALF GM values were significantly higher in invasive infections compared with non-invasive infections (1.49, IQR 0.30–7.85 vs 0.14, IQR 0.10–0.27; *P*-value = 0.007). Only 39 samples (7.2%) had a concurrent serum GM measurement, and only two yielded ODI values ≥0.5. Both of these were associated with pneumonia, caused by *Aspergillus terreus* and *Aspergillus flavus*, respectively (Table 3).

**TABLE 3:** GM ODI in TBA, BALF, and serum according to the presence of an *Aspergillus* infection^*a*^

		No *Aspergillus* infection	*Aspergillus* infection	*P* value	Non-invasive infection	Invasive infection	*P* value
BALF GM	*n* (%)	523 (96)	22 (4)		10 (45.5)	12 (54.5)	
	Median ODI (IQR)	0.07 (0.05–0.11)	0.27 (0.14–1.53)	<0.001	0.14 (0.10–0.27)	1.49 (0.30–7.85)	0.007
TBA GM	*n* (%)	523 (96)	22 (4)		10 (45.5)	12 (54.5)	
	Median ODI (IQR)	0.12 (0.07–0.21)	5.01 (2.27–10)	<0.001	3.83 (0.63–4.48)	6.32 (3.97–10)	0.065
Serum GM	*n* (%)	30 (76.9)	9 (23.1)		3 (33.3)	6 (66.7)	
	Median ODI (IQR)	0.07 (0.04–0.1)	0.12 (0.06–0.28)		0.06 (0.05–0.09)	0.14 (0.10–0.62)	

^*a*^ BALF, bronchoalveolar lavage fluid; TBA, tracheobronchial aspirate; GM, galactomannan; IQR, interquartile range; ODI, Optical Density Index.

**Fig 3:**
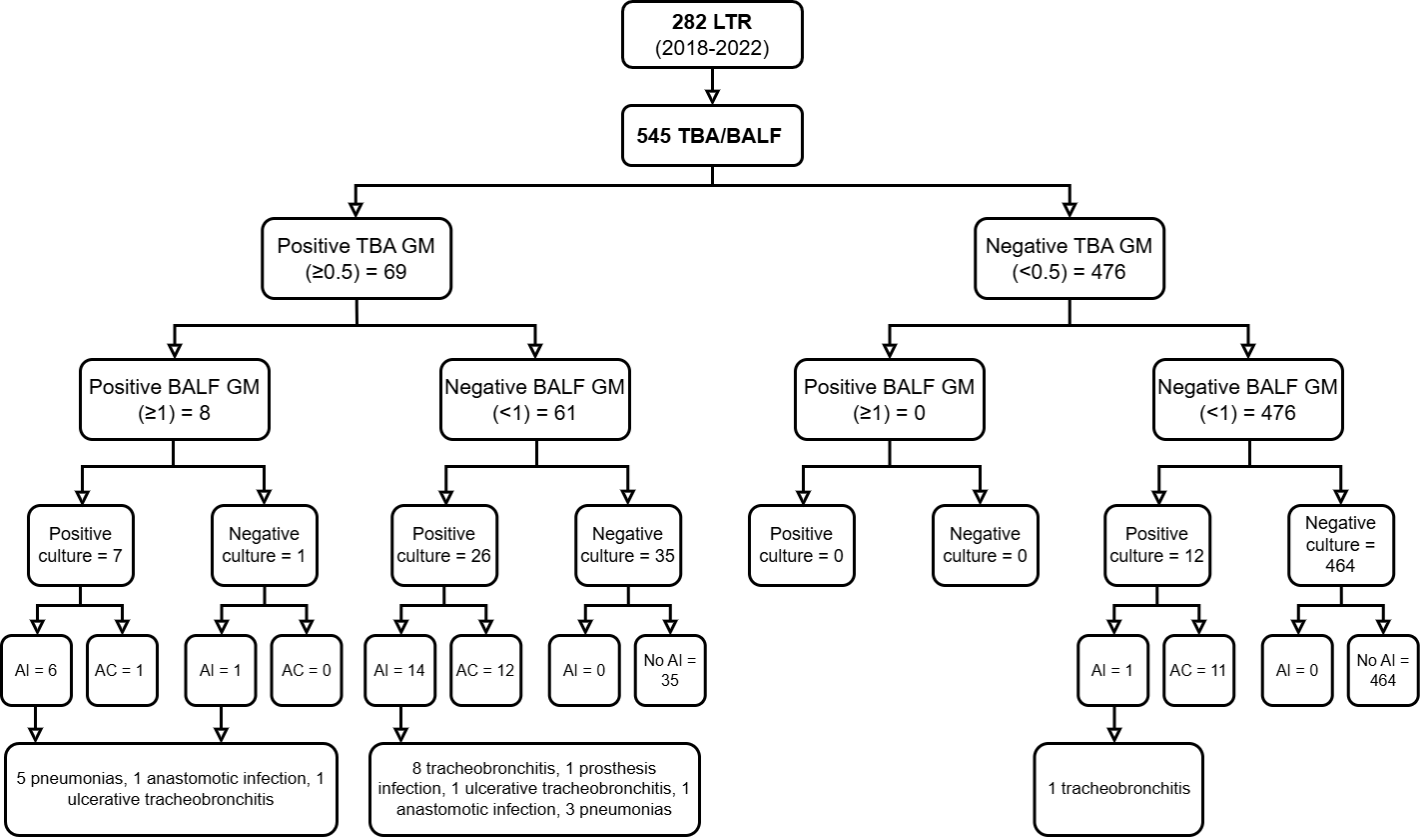
Classification of *Aspergillus* infection according to TBA GM, BALF GM, and fungal culture. Abbreviations: AC, *Aspergillus* colonization; AI, *Aspergillus* infection; BALF, bronchoalveolar lavage fluid; GM, galactomannan; LTR, lung transplant recipients; TBA, tracheobronchial aspirate.

Positive TBA GM results were observed in 21 of 22 AI using the ≥0.5 cut-off. Only one case was not classified as infection combining both TBA and BALF GM, which was an *Aspergillus niger* tracheobronchitis with mucopurulent plaques on bronchoscopy and no alternative diagnosis (0.26 TBA and 0.10 BALF GM ODI values). The participant received a 3-month course of isavuconazole, with complete resolution of symptoms. One participant had a positive BALF GM result with negative fungal culture, in which probable *Aspergillus* pneumonia was suspected, and empirical isavuconazole therapy was initiated. TBA and BALF GM values were 2.92 and 1.45, respectively; however, histopathology was negative for proven fungal infection, and the patient died from respiratory insufficiency (Fig. 3).

TBA and BALF GM measurements demonstrated moderate correlation (Pearson’s *r* = 0.536; 95% CI, 0.47–0.59; *P* < 0.0001), Fig. S1 Using the ≥0.5 ODI cut-off for TBA and ≥1 for BALF, agreement between the two tests was 88.8%. When combined, concordant TBA and BALF GM results (*n* = 484) achieved 87.5% sensitivity, 99.8% specificity, 416.5 and 0.13 positive and negative LR, respectively, and 99.6% accuracy for AI detection.

## DISCUSSION

Our findings suggest that TBA GM offers a valuable diagnostic alternative to BALF GM for AI in LTR. Positive or negative results showed strong evidence for or against the presence of AI. Importantly, combining TBA and BALF GM results improved diagnostic performance.

Proximal airway samples may better reflect early bronchial forms of aspergillosis which are seen in LTR, compared with hematologic patients. Specifically, the ongoing neovascularization of the bronchial tree and the absence of profound neutropenia may reduce angioinvasion, producing a prolonged airway phase (25, 26). The presence of hyphae can cause tissue necrosis and pyogranulomatous infiltrates, leading to tracheobronchitis and eventual ulceration (27). This is particularly notable in bronchial anastomosis, where perfusion is decreased (28). Even when infection reaches the distal airway, *Aspergillus* spp. may still be detectable proximally. Accordingly, higher GM values were detected more frequently in TBA than BALF, in parallel with a higher culture positivity rate in the proximal lower airway. There was also a higher recovery of yeasts and non-*Aspergillus* molds, reflecting potentially higher rates of fungal colonization in these samples. However, TBA GM correctly classified most cases despite this. Other studies have also supported a higher diagnostic yield for *Aspergillus* infection using proximal samples in non-LTR and non-neutropenic individuals (19, 24, 29).

On the other hand, BALF GM showed low sensitivity: 42.9% and 33.3% with ≥0.5 and ≥1 ODI cut-offs, respectively, although specificity remained high. Previous studies in LTR, using short-term universal prophylaxis, targeted prophylaxis, or preemptive therapy, reported sensitivities above 60% with a ≥1.0 ODI threshold, and proposed optimal cut-offs ranging from 0.5 to 1.5 ODI (10, 13–17). Importantly, in studies reporting the time to BALF, most AI occurred when patients were not receiving antifungal prophylaxis, which limits comparison with our cohort (13–15, 17, 18).

A higher incidence of non-invasive infections in our cohort, such as non-ulcerative tracheobronchitis or bronchial stent infection, could lower the sensitivity of BALF GM as the infection may still not have reached the distal respiratory tree at that stage. This was supported with higher values of BALF GM found in invasive AI compared to non-invasive. Some reports also suggest *Aspergillus fumigatus* may exhibit higher GM levels than *Aspergillus flavus* (30). As those are predominant in a setting with n-LAB prophylaxis, this may also impact GM detection. Only in one case *Purpureocillium lilacinum* isolation without *Aspergillus* spp. appeared to increase GM value, which has been reported previously (31, 32). Other molds such as *Penicillium* sp. were not found to increase the GM values in this study. Finally, serum GM was not consistently measured in this cohort, and only two cases of invasive infection showed positive results, suggesting that it may support the diagnosis of an angioinvasive form of AI in LTR in selected cases.

These data provide evidence for a possible alternative approach for AI diagnosis in LTR. When obtaining BALF is not feasible, a proximal airway sample could be used to reject the diagnosis of AI, or triggering AI suspicion and guiding antifungal therapy while waiting for fungal culture or a better condition to obtain BALF. Although TBA collection performed without bronchoscopy might yield comparable results, this cannot be confirmed by the present study. When BALF can be performed, a combined approach using both samples could improve the diagnostic performance. Concordant positive results in TBA and BALF yielded a dramatic increase in predictive value while maintaining high sensitivity and specificity.

There are several limitations in this study. First, the retrospective single-center design may present potential biases in data collection and limit the study’s applicability to other settings. Although a reasonable number of paired samples were included, only a few AI were detected, limiting the statistical power of the results within this subgroup. Second, our results could be affected by n-LAB lifelong prophylaxis and may decrease its generalization. Third, cases where BALF collection was not feasible, such as critically ill patients, may be underrepresented. The presence or absence of symptoms at the time of sample collection was not recorded, except in cases labeled as AI with positive microbiology; therefore, the indication of the bronchoscopy could not be evaluated in this study. However, pairing the samples obtained during the same procedure strengthened the comparative analysis between TBA and BALF. Finally, while diagnostic accuracy was evaluated, the clinical impact of how TBA GM results might change patient management or outcomes should be tested in further studies.

In conclusion, in this proof-of-concept study, TBA GM demonstrated higher sensitivity than BALF GM for detecting aspergillosis in LTR, with comparable specificity. Proximal airway sampling may serve as a useful diagnostic tool, either alone or in combination with BALF GM to enhance diagnostic accuracy. Prospective multicenter studies are needed to validate these findings and define the role of TBA GM in future diagnostic algorithms.

## AFTER EPUB

*[This article was published on 30 April 2026 with a funding source missing from Acknowledgments. The Acknowledgments were updated in the current version, posted on 10 June 2026.]*

## SUPPLEMENTAL MATERIAL

Supplemental material jcm.01556-25-s0001.docx Fungal isolation in culture in TBA and BALF, and correlation between TBA and BALF.

ASM does not own the copyrights to Supplemental Material that may be linked to, or accessed through, an article. The authors have granted ASM a non-exclusive, world-wide license to publish the Supplemental Material files. Please contact the corresponding author directly for reuse.
