# Supplementary material for: Diagnostic value of galactomannan in tracheobronchial aspirate for Aspergillus infection in lung transplant recipients (the GALACTBAS study)
Source: J Clin Microbiol. 2026 Apr 30;64(6):e01556-25. doi: 10.1128/jcm.01556-25 (PMC13251414; doi:10.1128/jcm.01556-25)
Supplement: Supplemental material — Fungal isolation in culture in TBA and BALF, and correlation between TBA and BALF. [file jcm.01556-25-s0001.docx]

**Supplementary materials**

Table 1 suppl: Fungal isolation in culture in TBA and BALF.

|  | Fungal isolation | *Aspergillus* | Other molds | Yeasts |
| --- | --- | --- | --- | --- |
| TBA | 131/545 (24%) | *Aspergillus terreus* (11)  *Aspergillus fumigatus* (10)  *Aspergillus flavus* (9)  *Aspergillus niger* (3)  *Aspergillus* *lentulus* (2)  *Aspergillus* *alliaceus* (1)  *Aspergillus* sp. (2) | *Lomentospora prolificans* (7)  *Penicillium* sp. (5)  *Purpureocillium lilacinus* (3)  *Scedosporium apiospermum* (3)  *Scopulariopsis/Microascus* sp. (3) | *Candida albicans* (44)  *Candida tropicalis* (8)  *Candida glabrata* (6)  *Candida parapsilosis* (5)  *Candida krusei* (3)  *Candida lusitaniae* (2)  *Candida guilliermondii* (1)  *Candida duobushaemulonii* (1)  *Blastobotrys* sp. (1)  *Saccharomyces cerevisiae* (1) |
| BALF | 54/545 (9.9%) | *Aspergillus* *terreus* (7)  *Aspergillus* *fumigatus* (3)  *Aspergillus* *flavus* (8)  *Aspergillus* *niger* (1)  *Aspergillus* *lentulus* (2) | *Lomentospora prolificans* (4)  *Penicillium* sp. (1)  *Purpureocillium lilacinus* (4)  *Scedosporium apiospermum* (1) | *Candida albicans* (15)  *Candida glabrata* (3)  *Candida parapsilosis* (2)  *Candida tropicalis* (2)  *Saccharomyces cerevisiae* (1) |

Figure 1 suppl: correlation between TBA and BALF.


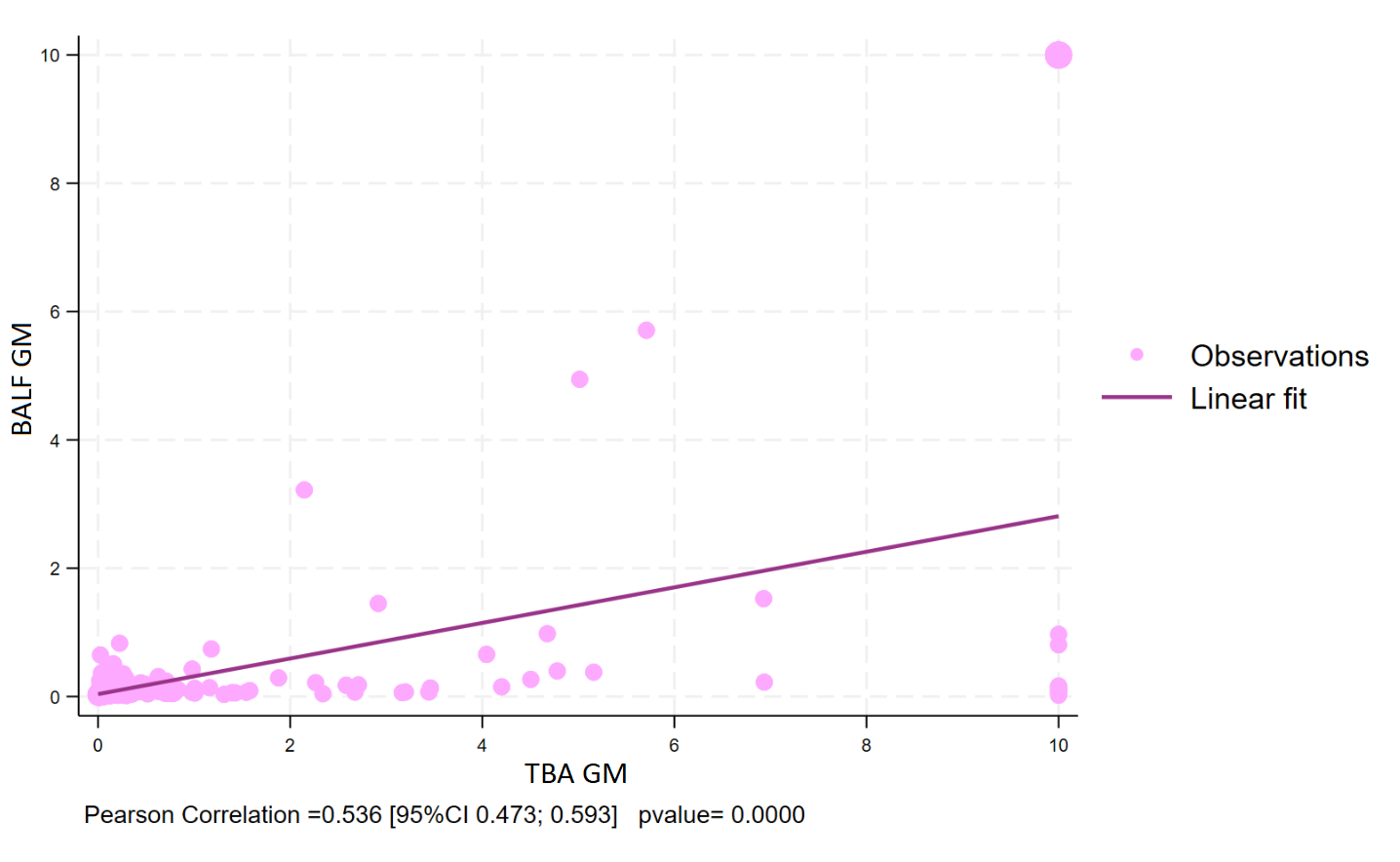


Abbreviations: BALF, bronchoalveolar lavage fluid; GM, galactomannan; TBA, tracheobronchial aspirate.
